# Clinical and Neurodevelopmental Characteristics of *Enterovirus* and *Parechovirus* Meningitis in Neonates

**DOI:** 10.3389/fped.2022.881516

**Published:** 2022-05-20

**Authors:** Silvia Bucci, Luana Coltella, Ludovica Martini, Alessandra Santisi, Domenico Umberto De Rose, Livia Piccioni, Francesca Campi, Maria Paola Ronchetti, Daniela Longo, Giulia Lucignani, Andrea Dotta, Cinzia Auriti

**Affiliations:** ^1^Department of Neurosciences, “Bambino Gesù” Children's Hospital Scientific Hospitalization and Treatment Institute (IRCCS), Rome, Italy; ^2^Department of Microbiology and Virology, “Bambino Gesù” Children's Hospital Scientific Hospitalization and Treatment Institute (IRCCS), Rome, Italy; ^3^Medical and Surgical Department of Fetus-Newborn-Infant, “Bambino Gesù” Children's Hospital Scientific Hospitalization and Treatment Institute (IRCCS), Rome, Italy; ^4^Department of Imaging, “Bambino Gesù” Children's Hospital Scientific Hospitalization and Treatment Institute (IRCCS), Rome, Italy

**Keywords:** neurodevelopment, outcomes, viral meningitis, viruses, newborns, infants

## Abstract

**Background:**

Non-polio-enteroviruses (EV) and human parechoviruses (HPeV) are small RNA viruses, which in newborns cause infections with a wide range of severity. Today molecular biology tools allow us to diagnose viral meningitis in neonates, sparing patients from useless antibiotics. Data on neurodevelopmental outcome of children who contract enterovirus meningitis in early childhood are still limited in the literature.

**Aims:**

To evaluate the neurodevelopmental outcome of newborns with documented e*nterovirus* and *parechovirus* meningitis contracted within the first months of life.

**Methods:**

*Enterovirus* and *parechovirus* were detected on cerebrospinal fluid (CSF) and plasma by RT-PCR. The virological typing was done according to WHO recommendations. During the hospitalization each neonate underwent many diagnostic and instrumental examinations, to evaluate any neurological lesions attributable to the infection. After the discharge children entered in an outpatient interdisciplinary assessment process, comprehensive of the administration of Bayley III scales up to 12 months old.

**Results:**

We observed longitudinally 30 children, born at term (mean GA 39.7 ± 0.8 weeks, mean birthweight was 3,457 ± 405 grams), who contracted *enterovirus* and *parechovirus* meningitis within the first month of life (mean age at diagnosis was 15.8 ± 7.33 days). We were able to perform the genetic typing only on 15/30 (50.0%) cerebrospinal fluid (CSF) samples from 15 neonates. We found MRI anomalies in 9/26 observed neonates (34.6%): one of them presented brainstem abnormality that are specific of enteroviral central nervous system (CNS) involvement. During the follow up children displayed an overall normal neurodevelopment and no deficit in visual and hearing areas. The mean cognitive (105.19 ± 8.71), speech (100.23 ± 8.22) and motor (97.00 ± 8.98) composite scores, assessed by Bayley III, were normal in 29/30 (96.7%). Despite this, children with pathological brain magnetic resonance imaging (MRI) scored significantly lower (*p* = 0.01) than children with normal brain MRI on cognitive subscale at 12 months of life.

**Conclusions:**

Early enterovirus infections can be associated to brain MRI abnormalities, more frequently the earlier the infection. Although within a normal range, our children with pathological brain MRI scored significantly lower than those with normal brain MRI on cognitive subscale at 12 months of life.

## Introduction

Non-polio *enteroviruses* (EV) and human *parechoviruses* (HPeV) are small RNA viruses, both within the family of *Picornaviridae*, causing frequently infections in the neonate (1).

*Enteroviruses*' capsid proteins (VP1, VP2, VP3, and VP4) determine the antigenicity and cellular penetration capacity of the virus and identify the serotype. *Enteroviruses* were traditionally divided into subgroups, based on their replication properties in cell cultures or animal models: they included *polioviruses* (PV), *coxsackie A* (CVA) *and B* (CVB), and *echoviruses* (ECHO for Enteric Cytopathogenic Human Orphan). The subsequently identified enteroviruses were defined by numbers (EV 68–71) (2). *Parechoviruses* were firstly labeled as *echoviruses* 22 and 23; now they have been reclassified and currently constitute HPeV genotypes 1 and 2. To date, 16 HPeV types have been identified (2). Enteroviral infections are transmitted by the fecal-oral and respiratory routes and mainly circulate in summer and autumn in temperate climates (3). Perinatal transmission is well-documented and occurs intrapartum (exposure to blood and/or maternal genital secretions) or postpartum (fecal-oral and/or respiratory samples). Intrauterine and ascending transmission are possible, but less common, and transmission of non-polio HEVs through breast milk has been hypothesized (4). The average incubation period is about 3–6 days for non-polio EVs (except for acute hemorrhagic conjunctivitis with 24–72 h), whereas the HPeV incubation period is unknown still now. The elimination of EVs and HPeVs persists for 1–3 weeks from the respiratory tract and for weeks or months in the stool; both can survive on environmental surfaces for several days (1).

Studies on neonates, especially concerning HPeV infections, are usually based on small case-series or case report, reflecting the lack of knowledge on their circulation in the neonatal age (5–10). In the United Kingdom, the combined incidence of EV and HPeV meningitis in neonates is 0.79/ 1,000 live births and 0.04/1000 live births, respectively, as recently estimated in an elegant study by Kadambari et al. (11). It appears more than double than bacterial meningitis and <1% of infected infants present complications or death attributable to the infection. The infection in neonates may present a wide range of severity: from pauci-symptomatic forms to meningitis with clear cerebrospinal fluid (11, 12), up to severe acute hepatitis with high mortality rates, especially in those born preterm (13).

The main risk factor for neonatal infection reported in the literature is the absence or the low titer of neutralizing antibodies in the mother. Risk factors for serious illness are maternal exposure, preterm birth and the onset of symptoms in early life (14). Severe illness can occur in infants infected with specific types of EVs (including echovirus 11 and *coxsackievirus B5*) and HPeV (such as *Human Parechovirus* 3), because their neurological tropism and virulence (15, 16).

Neonatal case series reported in the literature are scarce and knowledges on the long-term outcomes of enteroviral meningitis contracted during the neonatal age are poor (11, 17, 18).

We evaluated the neurological development of a group of children with documented *Enterovirus* and *Parechovirus* viral meningitis contracted within the first month of life. We aimed to determine any physical growth deficit, sensory defects, developmental, cognitive, prelinguistic and motor deficit during the 12-months follow-up of these babies.

## Methods

### Population

During current clinical activity we prospectively collected all clinical and laboratory data relating to newborns hospitalized with defined enterovirus meningeal infection and followed up 12 months at the outpatient service of Bambino Gesù Children's Hospital (Rome, Italy) from 2015 to 2021. Data of patients were obtained from medical records.

According to our neonatal department protocols, all infants admitted to the Neonatal Intensive Care Unit (NICU) with fever undergo contact isolation and laboratory sepsis work-up: complete blood counts, blood chemistry tests, blood culture, urinalysis, urine culture, C-reactive protein (CRP), procalcitonin (PCT), rectal swab for multidrug-resistant bacteria, nasal swab for *Staphylococcus aureus*. The lumbar tap is routinely performed as part of the initial sepsis work-up in all febrile neonates and infants. We examine the cerebrospinal fluid (CSF) samples for routine cell counts and chemistry and culture. We also performed real-time polymerase chain reaction (RT-PCR) on CSF, to detect the genome of viruses that most frequently cause meningoencephalitis in the newborn, such as *herpes virus* 1–2–6*, cytomegalovirus, enterovirus and parechovirus*.

### Definition of Viral Meningitis

Viral meningitis was defined on the basis of two or more of the following clinical signs (fever > or = 38°C, irritability, crying exhaustion, skin marbling, presence of skin rash, abdominal distension, diarrhea, poor feeding, bulging of anterior fontanel), associated with RT-PCR positivity of CSF and/or blood for RNA enteroviruses, and a CSF culture negative for bacteria. The clinical presentation of *parechovirus* central nervous system (CNS) infection is similar to that of enteroviral CNS infection, but with RT-PCR positivity of CSF and/or blood for *parechovirus*.

Although CSF pleocytosis is commonly absent in infants under 30 days of age in EV-positive infants (19), its presence may be observed in about half of cases of EV meningitis (20). Therefore, we also collected data on the CSF pleocytosis.

### Virological Testing

Nucleic acids were extracted from 400 μl of CSF and plasma samples, using the automatic platform QIAsymphony (Qiagen) and the Virus/Pathogen Midi kit (Qiagen), according to manufacturer's instructions. RT-PCR was performed on 7,900 HT Real Time PCR System (Applied Biosystems) with Enterovirus R-gene Argene (Biomérieux) or Parechovirus R-gene (Biomérieux), targeting the highly conserved sequences of the terminal 5' non-coding region of Enteroviruses or Parechoviruses, according to manufacturer's instructions.

Enterovirus RNA positive samples, after the extraction step, were submitted to sequencing analysis of part of the VP1 genomic region, coding for one of the capsid proteins, according to WHO recommendations (21).

The targeted region of the viral RNA sequence was reverse transcribed to single stranded complementary DNA (cDNA) via primer extension with the Multiscribe Reverse Transcriptase enzyme (Thermofisher Scientific) on Gene Amp PCR System 9,700 (Applied Biosystem), using four different primers.

Amplifications were carried on with AmpliTaq GoldTM DNA Polymerases (Applied Biosystem) on Gene Amp PCR System 9,700, using two sets of primers.

Sequencing reaction was set on with AN89 and AN88 primers and the Big Dye terminator v3.1 ready reaction mix (Life Technologies). Sequencing analysis was carried on ABI PRISM 3130 XL Genetic Analyzer (Applied Biosystem) according to manufacturer's conditions.

The sequences obtained were then compared with the published prototype sequences to identify unknown enteroviruses through comparison of partial VP1 sequence data (22).

### Management of Patients With Viral Meningitis During Hospitalization

In our Neonatal Intensive Care Unit all infants with meningitis of any etiology are monitored with amplitude-integrated electroencephalogram (aEEG) in the first 48–72 h of infection. Then the monitoring is suspended if the newborn improves and there are no suspected tracing anomalies. In case of suspected anomalies of the aEEG, or if the aEEG instrument is not available, an electroencephalogram (EEG) is performed, to rule out seizures. Immediately after carrying out the microbiological cultures, we start empiric antibiotics. If the diagnosis is of enteroviral meningitis, we suspend antibiotics and start immunoglobulins intravenously for three days, according to our local protocol. All of the infants described in this report followed this path of diagnosis and therapy.

### Neuroimaging

In our Unit all neonates with enteroviral meningitis undergo brain ultrasound (US) during the hospitalization. In newborns enrolled in this study the MRI examination was performed only in 26/30 (86.7%) newborns, as the protocol for the management of viral meningitis has changed over time. In the past MRI on neonates with viral meningitis was performed as a second level exam, if the brain US examination was pathological. The introduction of the mattress technique for brain MRI currently makes it possible to extend the examination to newborns with normal US, to study them better. The mattress technique allows to avoid sevoflurane sedation, that we administer in order to acquire MRI images with a good quality, only in the case of the child's restlessness. To date we did not observe any side effects. For this procedure we always require the informed consent of the patients' parents. MRI exam is generally performed within 7–15 days after the onset of the infection by 3 T scanner (MAGNETOM Skyra, Siemens, Erlangen, Germany).

### Follow-Up at 12 Months

After discharge from the NICU, children enter in an outpatient multidisciplinary follow-up, with quarterly clinical and instrumental assessments up to 12 months of age. All infants of this cohort were regularly evaluated to early intercept the presence of a neurological disability: the presence of a cerebral palsy (CP) was defined according to Bax et al. (23). To evaluate neurodevelopmental outcomes, we used the Bayley Development Scale for Toddlers and Infants Third Edition (BSDI-III, 2006) (24). This scale provided scores for three major development domains: cognition, language, and motor. Scores between 85 and 115 indicate normal development, while scores below 85 (-1 SD) indicate a developmental delay in the domain evaluated. The assessments were administered to patients by a developmental psychologist (S.B.), trained in BSID test procedures. The examiner was blinded to the patients' MRI findings and neonatal course. The psychologist assessed neurodevelopmental outcomes until 12 months. Children with scores within the normal range in all three domains were considered normal; children with a score below 85 (-1 SD) in at least one of the three domains were considered affected by a neurodevelopmental delay (25).

Visual function and retinal diseases were assessed by repeated examinations of the fundus and functional tests (electroretinogram–ERG—and visual evoked potentials –VEP—when necessary). Normal vision was defined as the “absence of any detectable pathology of the visual system”; mild abnormal vision as “the presence of a mild impairment which allowed useful vision”, and severe visual impairment as “a child functionally blind or perceives light only” (26).

Hearing function was explored by brainstem auditory evoked potentials (BAEPs). Auditory global function was defined as normal in “absence of any detectable pathology”, as mild if requiring hearing aids, or as severe if functionally deaf (uncorrected even with aids) (27).

### Statistical Analysis

Data are shown as numbers and percentages for categorical variables, whereas continuous variables are expressed as mean ± standard deviation (SD). Pearson correlations were performed to investigate the relationship between the results of the Bayley III composite score (cognitive, language, and motor) and the clinical characteristics of the infants' diagnosis at admission. Neurodevelopmental assessment in children with normal and pathological MRI were compared using ANOVA. Multiple logistic regression analysis was used to determine the most important predictors of pathological MRI. The odds ratio and 95% confidence interval for each variable were calculated. A *p* < 0.05 was considered statistically significant. Statistical analysis was performed using software SPSS (version 20 for Windows).

## Results

### Clinical Characteristics and Course

We longitudinally observed 30 children, full-term born, who contracted *enterovirus* and p*arechovirus* meningitis within the first month of life (mean age at diagnosis was 15.80 ± 7.33 days). Mean gestational age was 39.7 ± 0.8 weeks and mean birthweight was 3,457 ± 405 grams. Viral genome typing was performed on 15/30 (50.0%) neonates. *Echoviruses* (7, 9, 11, 18, 20 and 30) and *coxsackie v*iruses (A9 and B5) RNA were detected in 12/15 cerebrospinal fluid samples while *parechovirus* RNA on 3/15 samples. Two of those three babies with *parechovirus* meningitis showed abnormal brain MRI.

The Table 1 shows main clinical characteristics of our population of neonates and their length of hospital stay.

**Table 1:** Clinical characteristics and length of hospital stay of neonates with viral meningitis.

**Clinical characteristics of enrolled children**	***n* = 30**
Gender (males) [*n* (%)]	17 (56.7)
Gestational age (week) (mean ± SD)	39.7 ± 0.8
Birth weight (g) (mean ± SD)	3457 ± 405
Cesarean section [*n* (%)]	10 (33.3)
Apgar 5' (mean ± SD)	10 ± 0.2
Age on NICU admission (days) (mean ± SD)	15.8 ± 7.33
**Symptoms [*****n*** **Yes (%)]**	
Fever	30 (100)
Irritability	11 (36.7)
Poor feeding	7 (23.3)
Complaining behavior	7 (23.3)
Diarrhea	2 (6.7)
Rhinitis	2 (6.7)
Rush	1 (3.3)
Marbled skin	1 (3.3)
Exhaustible crying	1 (3.3)
Nuchal stiffness	1 (3.3)
Hyporeactivity	1 (3.3)
Length of hospital stay (mean ± SD)	10.41 ± 5.21

The most frequent symptom at the onset of the infection (100% of neonates) was high fever, even up to 39.2°C, associated with marked irritability during examination procedures and poor feeding.

All infants underwent aEEG monitoring, without seizure finding. Seizures were also ruled out by EEG in a total of seventeen infants (54.8%) with dubious aEEG trace.

One of those children with a human herpesvirus 6 (HHV6) coinfection, had epileptic anomalies on the electroencephalographic trace, in particular low waves in the parieto-temporal site and a poor background organization. The diagnosis of epilepsy was never made by neurologists, the EEG trace progressively improved, and anticonvulsant therapy was never necessary. Currently this child is fine and presents a normal neurodevelopmental outcome. Due to the presence of HHV6 co-infection we started therapy with intravenous Ganciclovir and then with oral Valganciclovir.

In summary, 29/30 patients (96.7%) had EEG and/or aEEG trace with no signs of seizures.

### Characteristics of the Cerebrospinal Fluid

In 55% of our infants we found an alteration of the CSF. Of these, 32% had frankly pathological liquor, with a predominance of mononuclear cells. The three infants with *parechovirus* meningitis had CSF with less than 5 white blood cells and normal levels of protein and glucose. Pleocytosis was not associated with a more serious course of acute disease nor with worse long-term outcomes. Table 2 shows the CSF characteristics of our patients.

**Table 2:** Characteristics of CSF in neonates with meningitis.

**Chemical characteristics**	**n = 30**
**CSF appearance [*****n*** **(%)]**	
Clear	26 (86.7)
Almost clear	2 (6.7)
Opalescent	2 (6.7)
**White blood cell count [n (%)]**	
Normal	13 (43.3)
Abnormal with monocyte prevalence	7 (23.3)
Pathological	10 (33.3)
**Protein level [*****n*** **(%)]**	
Normal	6 (20.0)
Pathological	24 (80.0)
**Glucose levels [*****n*** **(%)]**	
Normal	16 (53.3)
Pathological	14 (46.7)

*Normal values at our laboratory: CSF white blood cell count 0-3 cells/mm3; CSF protein level 58-150 mg/dl; glucose levels 32-82 mg/dl*.

### Biological Typing of Viruses

In all 30 enrolled patients we identified viral RNA. We were able to perform the genetic typing only on 15/30 (50.0%) CSF samples. In 9 of them we typed *echoviruses* (7,9,18,11,20,30): e*chovirus* 30 in three and *echovirus* 18 in two samples; the other virus typed were c*oxsackie* B5 in one patient's CSF and *coxsackie* A9 in two patients' CSF. Three infants had *parechovirus* meningitis (Supplementary Table 1). One infant with *enterovirus* meningitis had *HHV6* co-infection. The absence of viral DNA determined with the analysis of the child's hair bulb allowed us to exclude the chromosomal integration of the viral genome.

We did not isolate EV71, which seems to be the most neurotropic and the one most frequently involved in the genesis of brainstem encephalitis (28).

In our patient with brainstem lesions, we isolated *echovirus* 30, but such lesions were considered secondary to perinatal asphyxia, according to the radiological characteristics and the history of the patient.

### Neuroimaging

All 30 patients underwent brain ultrasound (US), while only 26 (86.7%) also performed brain MRI. The NICU protocol concerning viral meningitis has been modified over time. At the beginning the brain MRI was performed only in newborns with brain US abnormalities. Subsequently we decided to carry out the examination to all patients, having introduced the heated mattress technique, which avoids the sedation of the baby. We did not detect abnormalities in all newborns by brain ultrasounds. Conversely, we found non-specific brain abnormalities in 9/26 newborns (34.6%) by, which was performed on patients between 12 and 65 days of life (mean 38 ± 26 days). Among 9 neonates with MRI anomalies, dural contrast enhancement was found in 3/9 (33.3%) neonates (one of them with echovirus 20 infection); diffuse white matter alterations, probably due to delay of myelination, in 2/9 (22.2%) neonates (one had an infection by *echovirus* 7); cytotoxic oedema in 1/9 (11.1%) and periventricular microbleeds in 1/9 (11.1%) neonates (infected by *coxsackievirus* B5). Moreover, 1/9 (11.1%) neonate with *echovirus* 11 infection presented brainstem alterations and 1/9 (11.1%) with *echovirus* 30 showed mildly hyper-intense signals on T2-weighted images in the head of right caudate nucleus. Among the 17 patients with normal MRI one presented a subdural effusion, perhaps due to a difficult delivery, but the MRI was considered normal by neuro-radiologists. Analyzing data by logistic regression model (Table 3), pathological brain MRI was associated with age at admission to the NICU: the younger the infant, the greater the likelihood of brain MRI abnormalities (OR: 0.788; 95% CI: 0.634 −0.978; *p* = 0.031).

**Table 3:** Logistic regression models of MRI results.

**Predictor**	**eß (Odds ratio)**	**95% CI**	***p*-value**
Age at admission to the NICU (days)	0.788	0.634–0.978	0.031
Birth weight	1.003	0.999–1.006	0.106
CSF white blood cell count	1.001	0.995–1.007	0.725
CSF protein level	1.063	0.942–1.199	0.325
CSF glucose levels	1.144	0.945–1.384	0.168

### Follow-Up at 12 Months

During the clinical follow-up, children displayed normal weight growth rates. The neurodevelopmental assessment by Bayley-III is presented in Table 4. At 12 months of life we found that most babies (96.7%) had no sequelae on cognitive, language, and motor BSDI-III composite score results. None had a cerebral palsy. Only one child, with previous perinatal asphyxia, had a mild delay in fine and gross motor skills and in receptive language. All infants reported to have a normal vision and a normal hearing function.

**Table 4:** Neurodevelopmental outcomes of 30 neonates at 12 months of age.

**Neurodevelopmental assessment**	***n* = 30**
**Bayley-III scales (mean** **±SD)**	
Cognitive	105.19 ± 8.71
Language	100.23 ± 8.22
Motor	97.00 ± 8.98
Abnormal cognitive results, *n* (%)	0 (0)
Abnormal language results, *n* (%)	1 (3.3)
Abnormal motor results, *n* (%)	1 (3.3)

Correlations between Bayley III composite score (cognitive, language, and motor) and the clinical characteristics of the infants' diagnosis at admission are reported in Supplementary Table 2.

Children with pathological MRI during hospitalization scored significantly lower than children with normal MRI on cognitive Bayley III subscale at Neurodevelopmental assessment at one year of age, even if all the scores obtained fell within the normal range (*F* = 6.289; *p* = 0.022) (Table 5).

**Table 5:** Neurodevelopmental outcomes of 30 neonates at 12 months of age.

	**Normal MRI**	**Pathological MRI**	**F**	***p-*value**
Bayley III cognitive scale	109.55 ± 8.20	100.56 ± 7.68	6.289	0.022
Bayley III language scale	102.18 ± 8.38	99.33 ± 9.69	0.497	0.490
Bayley III motor scale	100.64 ± 8.03	95.25 ± 10.90	1.548	0.230

## Discussion

Viral meningitis is very common within 90 days of life. The frequency of diagnosis has increased many times over the past 10 years, thanks to the spread of molecular biology techniques. This phenomenon has been observed in all European countries (11).

A proper and early detection of etiology of viral meningitis by molecular biology could help to identify biological types associated with more severe conditions and monitor their associated disease burden and allows to spare the improper use of antibiotics (29, 30).

As in neonates at term and in young infants enteroviral meningitis usually has a benign course, it is not thoroughly studied, mainly with regard to long-term outcomes. Data on children affected after the acute phase of the disease and hospital discharge are scarce. The British Pediatric Surveillance Unit reports that only 38% (254/668) of children with EV and 46% (16/35) of children with HPeV meningitis were seen by hospital clinicians within 12 months of discharge from the ward, for an evaluation of the infection outcomes (11).

In this case series of 30 neonates we have found that 9/26 (34.6%) children, who became infected soon after birth presented pathological brain MRI during hospitalization. The difference of 10-12 days in carrying out the MRI does not change the prognostic value of this examination in meningitis as lesions, after the acute phase of the inflammatory injury, do not regress completely and show the same signal characteristics. These slight anomalies seem to be associated with a cognitive Bayley III score at 12 months, lower than in children with normal brain MRI, despite within the normal range. The linguistic and motor scores seem to be not affected. We found a mild delay in fine and gross motor skills and in receptive language only in one child, who suffered asphyxia at birth. It has been reported that *Enterovirus* encephalomyelitis has characteristic lesion located in the posterior portions of the brain stem, substantia nigra and dentate nucleus (31): in our cohort, only one of our patients presented brainstem alterations and at 2 years of age he still has still difficulties in language skills.

Kurz et al. described that some infants with *human parechovirus* (mainly genotype 3) could have a cerebral hemorrhage, but we did not find any hemorrhages; however, our HPeV infection cases have not been typed and it could be a milder genotype (32).

The available studies on neurological long-term outcomes in neonates with early meningeal enteroviral infection have been carried out on very small numbers of patients. The largest longitudinal follow-up study comes from the United Kingdom, carried out from July 2014 to July 2015 and included 668 cases in infants <3 months old and followed up till 12 months of life (11). In this case series, no child showed sensorineural deficits, three patients presented seizures, one myocarditis and one hypotonia of the lower limbs, with an estimated risk of long-term sequelae of 0.6% (4/666, 95% CI, 0.2–1.5%).

A systematic review and meta-analysis about *parechovirus* CNS infection presented an increasing proportion of children with neurological sequalae over time (33): conclusions seem in line with those presented in this report.

Furthermore, Van Hinsbergh et al. recently highlighted the importance of follow-up of these infants to detect subtle neurodevelopmental delay and start early interventions (34).

Studies reporting outcomes after viral meningitis were markedly heterogeneous in age of infection, length of follow-up, inclusion of case controls, and outcomes measured, making it difficult to compare data (17).

Limitations of this study include the single-center site and the small sample size. However, we evaluated infants with viral meningitis in the first month using always the same neurodevelopmental test. We confirm that most infants with EV and HPeV meningitis don't go toward marked linguistic and motor deficits but have a lower cognitive score than children with no brain MRI abnormalities, despite within normal ranges.

## Conclusions

Our study evidenced that early *enterovirus and parechovirus* infections can be associated with slight brain MRI abnormalities, more frequently the earlier the infection. Children with abnormal brain MRI during hospitalization may have long term cognitive Bayley III subscale score lower than children with normal brain MRI at the neurodevelopmental assessment. Despite the overall favorable outcome, children with *enterovirus and parechovirus* meningitis in the first month of life should undergo a multidisciplinary follow-up. It is important to carry out the early diagnosis of developmental defects in order to undertake early rehabilitation interventions.

## Data Availability Statement

The raw data supporting the conclusions of this article will be made available by the authors, without undue reservation.

## Ethics Statement

The studies involving human participants were reviewed and approved by Scientific Directorate, Bambino Gesù Children's Hospital, Rome. Written informed consent to participate in this study was provided by the participants' legal guardian/next of kin.

## Author Contributions

SB, CA, and LM conceptualized and designed the study, designed the data collection instruments, collected data, contributed to the interpretation of the results, reviewed, and revised the manuscript. LC and LP performed virology testing and revised the manuscript. DD performed literature search, designed the data collection instruments, collected data, analyzed data, and drafted the initial manuscript. AS and MR followed up patients and revised the manuscript. FC and AD collected data and revised the manuscript. DL and GL interpreted brain MRI and revised the manuscript. CA conceptualized and designed the study, supervised data collection, contributed to the interpretation of the results, reviewed, and revised the manuscript. All authors approved the final manuscript as submitted and agree to be accountable for all aspects of the work.

## Conflict of Interest

The authors declare that the research was conducted in the absence of any commercial or financial relationships that could be construed as a potential conflict of interest. The reviewer AB declared past co-authorships with one of the authors AD and the absence of any ongoing collaboration with any of the authors to the handling editor.

## Publisher's Note

All claims expressed in this article are solely those of the authors and do not necessarily represent those of their affiliated organizations, or those of the publisher, the editors and the reviewers. Any product that may be evaluated in this article, or claim that may be made by its manufacturer, is not guaranteed or endorsed by the publisher.
