# Supplementary material for: Clinical and Neurodevelopmental Characteristics of Enterovirus and Parechovirus Meningitis in Neonates
Source: Front Pediatr. 2022 May 20;10:881516. doi: 10.3389/fped.2022.881516 (PMC9165715; doi:10.3389/fped.2022.881516)
Supplement: Supplementary file 1 [file Data_Sheet_1.pdf]

SUPPLEMENTARY TABLES

Supplementary Table 1. Viral typing results on 15/30 neonates enrolled in the study.

**Typing**	**Patients (n)**
Echovirus 7	1
Echovirus 9	1
Echovirus 11	1
Echovirus 18	2
Echovirus 20	1
Echovirus 30	3
Coxsackie B5	1
Coxsackie A9	2
Parechovirus	3
Typing not performed	15
Total	**30**

Supplementary Table 2. Correlations between clinical characteristics of the infants' diagnosis at admission and Bayley III composite scores (cognitive, language, and motor)

	**1**	**2**	**3**	**4**	**5**	**6**	**7**	**8**	**9**
1. Age at meningitis admission (days)	1								
2. Duration of hospital stay (days)	0.11	1							
3. Gestational age	0.18	0.31	1						
4. Birth weight	0.12	0.12	**0.48****	1					
5. White blood cell count	-0.12	**0.46***	0.13	-0.23	1				
6. Protein level	**-0.38***	**0.49****	0.18	-0.12	**0.49****	1			
7. Glucose levels	0.23	-0.06	0.07	-0.22	-0.31	-0.16	1		

8. Bayley-III Cognitive	0.19	0.02	0.15	-0.18	0.13	0.06	-0.01	1	
9. Bayley-III Language	-0.10	0.10	**0.50***	0.18	0.03	0.27	-0.17	0.36	1
10. Bayley-III Motor	0.03	0.36	0.12	-0.22	0.04	0.29	0.01	0.30	**0.49***

** p <0.01; *p <0.05

Table S1 reports the Spearman's correlation coefficient between clinical characteristics of the infants' diagnosis at admission and Bayley III composite score (cognitive, language, and motor).

Variables on the x axis are the same as those on the Y axis which are reported only with a numeric code.

In example: the protein level (variable 6) correlates negatively and significantly with the age at which children contracted the infection (variable 1) (i.e. the lower the age at which they are sick higher the protein level, r = -0.38; p <0.05).
